# Lymphoblastoid cell lines as a model to understand amyotrophic lateral sclerosis disease mechanisms

**DOI:** 10.1242/dmm.031625

**Published:** 2018-03-01

**Authors:** Orietta Pansarasa, Matteo Bordoni, Lorenzo Drufuca, Luca Diamanti, Daisy Sproviero, Rosa Trotti, Stefano Bernuzzi, Sabrina La Salvia, Stella Gagliardi, Mauro Ceroni, Cristina Cereda

**Affiliations:** 1 Genomic and post-Genomic Center, IRCCS Mondino Foundation, 27100 Pavia, Italy; 2 Department of Brain and Behavioral Sciences, University of Pavia, 27100 Pavia, Italy; 3 General Neurology Unit, IRCCS Mondino Foundation, 27100 Pavia, Italy; 4 Department of Neurodiagnostics and Services, Laboratory of Clinicals and Chemicals Analysis (SMeL), IRCCS Mondino Foundation, 27100 Pavia, Italy; 5 Department of ‘Medicina Diagnostica e dei Servizi’, IRCCS Policlinico San Matteo Foundation, 27100 Pavia, Italy

**Keywords:** ALS model, LCLs, Protein aggregation, Mitochondria

## Abstract

In the past, amyotrophic lateral sclerosis (ALS) has been considered a ‘neurocentric’ disease; however, new evidence suggests that it should instead be looked at from a ‘multisystemic’ or ‘non-neurocentric’ point of view. From 2006, we focused on the study of non-neural cells: ALS patients’ peripheral blood mononuclear cells (PMBCs) and lymphoblastoid cell lines (LCLs). Here, we characterize LCLs of sporadic ALS (sALS) and patients carrying *SOD1*, *TARDBP* and *FUS* mutations to identify an ALS biologically relevant molecular signature, and determine whether and how mutations differentially affect ALS-linked pathways. Although LCLs are different from motor neurons (MNs), in LCLs we found some features typical of degenerating MNs in ALS, i.e. protein aggregation and mitochondrial dysfunction. Moreover, different gene mutations have different effects on ALS cellular mechanisms. *TARDBP* and *FUS* mutations imbalance mitochondrial dynamism toward increased fusion, whereas sALS and *SOD1* mutations mainly affect fission. With regards to protein aggregation and/or mislocalization, *TARDBP* and *SOD1* mutations show the presence of aggregates, whereas *FUS* mutation does not induce protein aggregation and/or mislocalization. Finally, all LCLs, independently from mutation, are not able to work in a condition of excessive energy request, suggesting that mitochondria from ALS patients are characterized by a significant metabolic defect. Taken together, these data indicate that LCLs could be a valid cellular model in ALS research in the identification and study of specific pathological pathways.

## Abstract (precis)

**Summary:** The identification of a patient-specific and non-invasive model is crucial in ALS research. Here, the authors present a reliable characterization of lymphoblastoid cell lines from both sporadic and mutated ALS patients.

## INTRODUCTION

Amyotrophic lateral sclerosis (ALS) is a complex multi-factorial and multi-systemic disorder characterized by massive motor neuron (MN) loss in the brainstem, spinal cord and motor cortex (Robberecht and Philips, 2013). Multiple pathways might play a critical role in MN survival, including glutamate-induced excitotoxicity, endoplasmic reticulum stress, proteasome inhibition, secretion of toxic factors by non-neuronal cells, oxidative stress, axonal disorganization, neuromuscular junction abnormalities, aberrant RNA processing, protein aggregation and mitochondria-mediated damage (Taylor et al., 2016).

Mitochondrial alterations represent a milestone in all the major neurodegenerative diseases, including ALS (Smith et al., 2017; Lin and Beal, 2006). The evidence that mitochondria are impaired in ALS pathogenesis is clear from various studies involving cellular and animal models as well as ALS patients (Tefera and Borges, 2017; Cacabelos et al., 2016; Allen et al., 2015). Defects in mitochondrial Ca^2+^ buffering ability and in the activity of the electron transport protein complex were reported in the spinal cord of mutant *SOD1* mice during the pre-symptomatic phase of the disease (Abu-Hamad et al., 2017; Nalbandian et al., 2015; Kawamata and Manfredi, 2010). Furthermore, defective mitochondria were identified in mutant FUS- and TDP-43-expressing cells and *Drosophila*, which display aberrant and non-functional mitochondria (Khalil et al., 2017; Onesto et al., 2016; Deng et al., 2015). Abnormal mitochondrial morphology was observed in neurons and peripheral cells of sporadic (sALS) and familial ALS (fALS) patients (Rodriguez et al., 2012); moreover, mitochondrial fragmentation has also been documented in ALS cell and animal models (Song et al., 2013). The morphology of the mitochondrial network is influenced by the delicate balance between two opposing events: fusion and fission (Itoh et al., 2013; da Silva et al., 2014). These two processes are highly coordinated and strictly controlled by specific proteins: dynamin-related protein1 (Drp1) and fission 1 (Fis1) for the fission process, and mitofusin 1/2 (MFN1/2) and optic atrophy protein 1 (OPA1) for the fusion process (Gao et al., 2017). Mitochondrial fragmentation is strictly dependent on changes in the expression of mitochondrial fusion and fission regulators in experimental models expressing ALS-associated mutant SOD1 (Song et al., 2013). Also, neurons expressing ALS-associated mutant TDP-43 and mutant FUS show mitochondrial fragmentation and alterations in fusion and fission regulators (Deng et al., 2015; Wang et al., 2013).

A common hallmark of neurodegenerative diseases is the presence of misfolded protein aggregates in affected regions of the nervous system (Cuanalo-Contreras et al., 2013). Conformational alterations due to protein misfolding may contribute to disease by either a gain of a toxic function or by the loss of proper biological activity of a protein. Moreover, the formation of abnormal protein aggregates can inhibit indispensable cellular functions (Takalo et al., 2013).

ALS has been historically considered a ‘neurocentric’ disease that primarily affects MNs. Recent evidence suggests that also non-neural cells, i.e. microglia, astrocytes, peripheral blood mononuclear cells (PBMCs) and skeletal muscle fibers, could participate in triggering MN degeneration (Robberecht and Philips, 2013; Cereda et al., 2013; Dobrowolny et al., 2008; Rossi et al., 2008). This new evidence deeply switches the ALS hypothesis from focusing on it as a ‘neurocentric’ disease to looking at the pathology from a ‘multisystemic’ or ‘non-neurocentric’ point of view. Starting from 2006, our group extensively focused its attention on the study of non-neural cells, specifically to patients' peripheral blood mononuclear cells (PBMCs) and lymphoblastoid cell lines (LCLs) (Cereda et al., 2013; Guareschi et al., 2012; Gagliardi et al., 2010; Cereda et al., 2006).

The present study aimed, for the first time, to extensively characterize LCLs from sALS and mutated patients to identify any biologically relevant molecular signature associated with ALS pathology. Finally, we aimed to highlight whether and how mutations in the *SOD1*, *TARDBP* and *FUS* genes differentially affect ALS-linked pathways.

## RESULTS

Increasing evidence suggests that peripheral tissues, such as PBMCs and LCLs, which have gained importance in ALS research, share some pathological features with degenerating MNs (Cereda et al., 2013; Guareschi et al., 2012; Gagliardi et al., 2010; Cereda et al., 2006). Here, we focused our attention on the characterization of LCLs in ALS; we investigated two of the main ALS-related pathogenic mechanisms: accumulation of protein aggregates and mitochondrial dysfunction.

### *SOD1*, *TARDBP* and *FUS* mutations showed alterations in soluble protein levels in LCLs

**Fig. 1.**
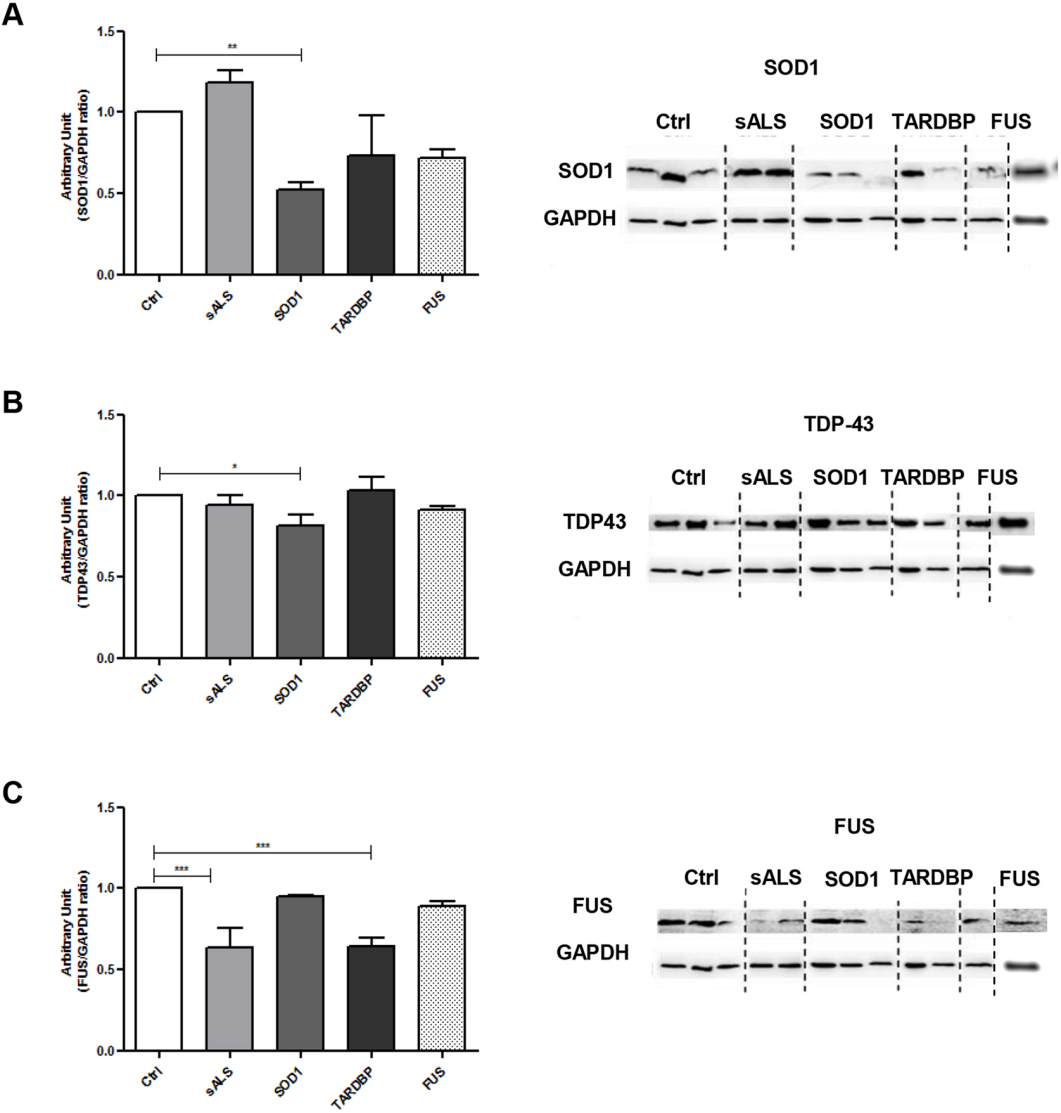
***SOD1*, *TARDBP* and *FUS* mutations lead to changes in soluble protein levels in LCLs.** (A-C) Representative immunoblots for total SOD1, TDP-43 and FUS protein expression levels in Ctrl, sALS, *SOD1*, *TARDBP* and *FUS* LCLs. LDH was used for sample normalization. Densitometric analysis of WB data are performed by ImageJ software. Statistical analysis was carried out on *n*=4 Ctrl, *n*=4 sALS, *n*=3 *SOD1*, *n*=2 *TARDBP* and *n*=2 and *FUS* LCLs. Data are means±s.e.m. and one-way ANOVA followed by Dunnett's multiple comparison test as a *post hoc* test. *P<0.05 in B, **P<0.01 in A and ***P<0.001 in C, all relative to Ctrl.

To verify whether *SOD1*, *TARDBP* and *FUS* mutations affected protein expression levels, western blotting (WB) for SOD1, TDP-43 and FUS have been performed on total soluble protein fractions (Fig. 1A-C). SOD1 protein expression levels showed an overall reduction in all patients with mutations versus healthy controls (Ctrl); only in patients with *SOD1* mutation when compared to Ctrl was this reduction statistically significant (*P*<0.01, Fig. 1A). Similarly, TDP-43 (encoded by *TARDBP*) protein expression levels showed a significant decrease (*P*<0.05) in *SOD1*-mutated patients versus Ctrl; no changes were instead reported in sALS, *TARDBP*- and *FUS*-mutated patients (Fig. 1B). With regards to the expression of FUS protein, we reported a significant decrease in sALS and *TARDBP*-mutated patients compared to Ctrl (*P*<0.001), whereas no differences were reported for patients carrying mutations in *SOD1* or *FUS* (Fig. 1C). We also evaluated mRNA levels for *SOD1*, *TARDBP* and *FUS*. We reported an increase in *SOD1* mRNA levels in *SOD1*-mutated patients, whereas no changes were reported for *TARDBP* and *FUS* mRNA levels in both sALS and mutated patients (Fig. S1A-C).

### *SOD1*, *TARDBP* and *FUS* mutations lead to altered protein localization in LCLs

It is widely accepted that the mislocalization of SOD1, TDP-43 and FUS proteins can ultimately account for various ALS pathological signaling (Ido et al., 2011; Ilieva et al., 2009). To disclose the molecular basis and the downstream cascades of the mislocalized and/or aggregated proteins, we fractionated nucleus and cytoplasm and evaluated SOD1, TDP-43 and FUS by WB and immunofluorescence in LCLs from Ctrl, sALS and mutated patients.

**Fig. 2.**
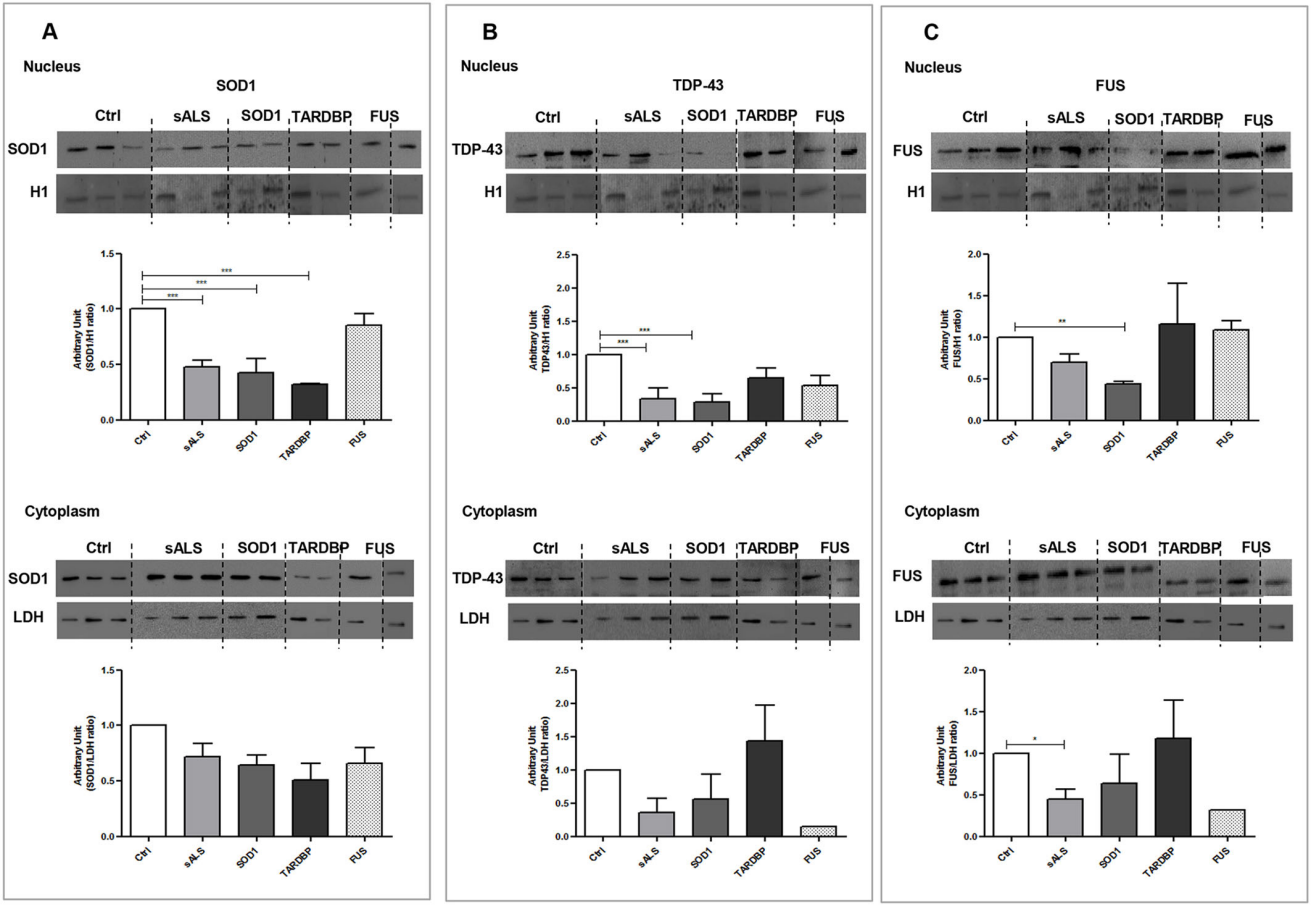
***SOD1*, *TARDBP* and *FUS* mutations showed protein relocalization in LCLs.** (A-C) Representative immunoblots for nuclear and cytoplasmic SOD1, TDP-43 and FUS protein expression levels in Ctrl, sALS, *SOD1*, *TARDBP* and *FUS* LCLs. Histone H1 was used for nuclear sample normalization; LDH was used for cytoplasm sample normalization. Densitometric analysis of WB data are performed by ImageJ software. Statistical analysis was carried out on *n*=4 Ctrl, *n*=4 sALS, *n*=3 *SOD1*, *n*=2 *TARDBP* and *n*=2 and *FUS* LCLs. Data are means±s.e.m. and one-way ANOVA followed by Dunnett's multiple comparison test as a *post hoc* test. *P<0.05 in C (cytoplasm), **P<0.01 in C (nucleus) and ***P<0.001 in A and B (cytoplasm), all relative to Ctrl.

WB results stated that SOD1 protein expression levels are reduced in cytoplasm (Fig. 2A) and decreased significantly in nucleus (*P*<0.001) of sALS, *SOD1*- and *TARDBP*-mutated patients versus Ctrl, suggesting that SOD1 did not re-localize from cytoplasm to the nuclear compartment (Fig. 2A).

In the nuclear compartment, WB results showed a decrease in TDP-43 protein expression levels in all ALS patients compared to Ctrl, which gained statistical significance in sALS and *SOD1*-mutated (*P*<0.001) patients (Fig. 2B). In the cytoplasm, we reported an increase in TDP-43 protein expression levels only in *TARDBP*-mutated patients compared to Ctrl (Fig. 2B); nevertheless, statistical significance was not reached.

Concerning FUS protein expression levels, no great changes were observed in the nuclear compartment except for *SOD1*-mutated patients, which showed a significant decrease compared to Ctrl (*P*<0.01, Fig. 2C). Instead, in the cytoplasm, we reported a significant reduction in FUS protein expression levels in sALS patients (*P*<0.05) compared to Ctrl (Fig. 2C).

### *SOD1*, *TARDBP* and *FUS* mutations show protein relocalization and aggregation in LCLs

**Fig. 3.**
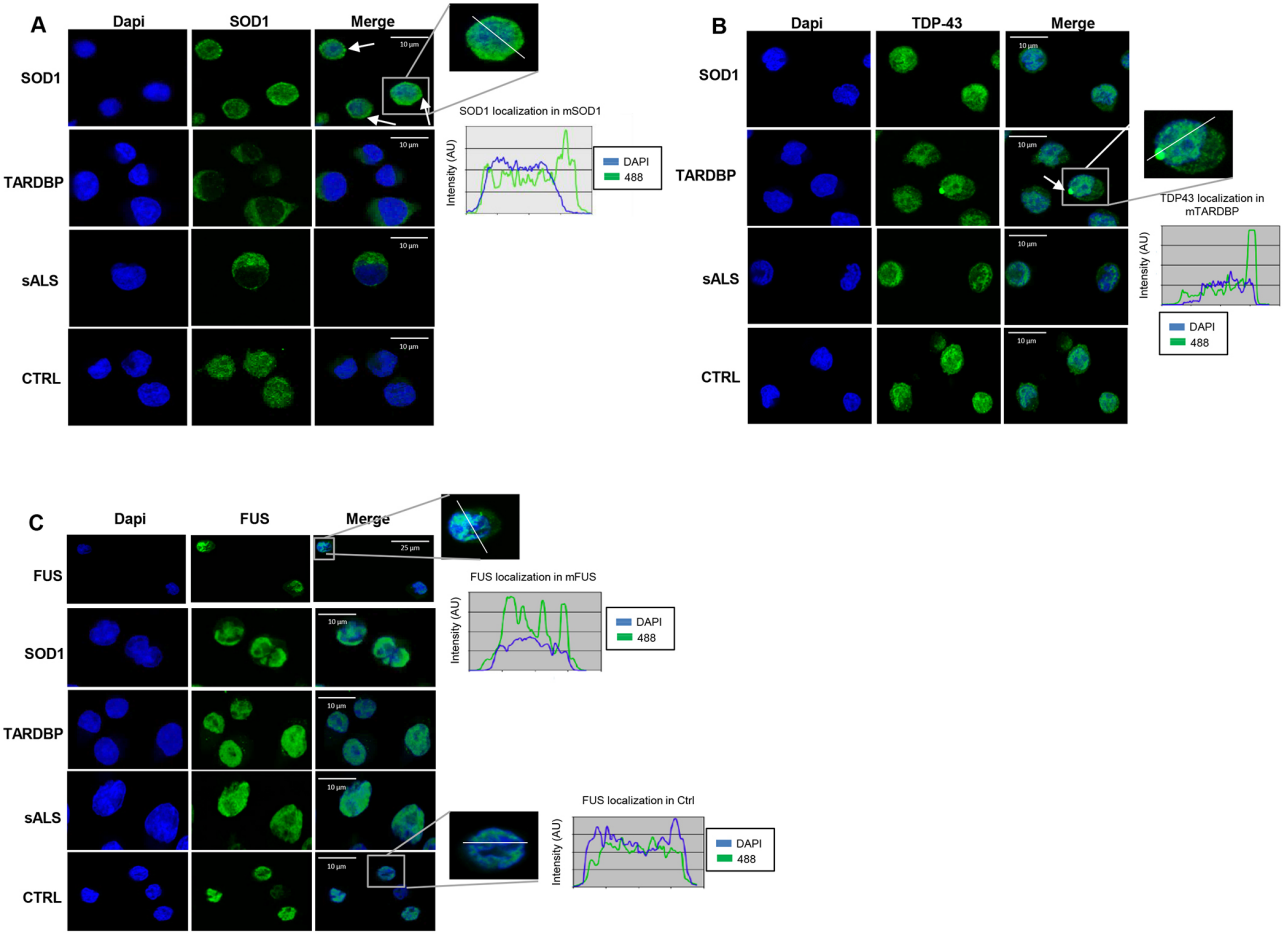
***SOD1*, *TARDBP* and *FUS* mutations showed protein relocalization and aggregation in LCLs.** Representative immunostaining of SOD1, TDP-43 and FUS in LCLs of sALS, *SOD1*-, *TARDBP*- and *FUS*-mutated patients. Immunostaining of SOD1 (A) showed a cytoplasmic homogeneous distribution in Ctrl, sALS and *TARDBP*-mutated patients; in *SOD1*-mutated patients, aggregates at the cytoplasm/perinuclear level were present (white arrows). TDP-43 immunostaining (B) showed the presence of cytoplasmic aggregates (see white line and intensity profile analysis) in *TARDBP*-mutated patients; Ctrl, sALS and *SOD1*-mutated patients presented a homogeneous distribution of TDP-43 staining. Immunostaining of FUS showed no cytoplasm mislocalization and/or aggregation of FUS protein (C).

In Fig. 3A, SOD1 immunostaining showed a cytoplasmic homogeneous signal distribution in Ctrl, sALS and *TARDBP-* mutated patients. Instead, in *SOD1*-mutated patients, we evidenced the presence of aggregates at the cytoplasm/perinuclear level; also, the intensity profile analysis (along the white line in Fig. 3A) showed an elevated peak right outside the nucleus, pointing at aggregate formation.

In *TARDBP*-mutated patients, TDP-43 immunostaining analysis also highlighted the presence of aggregates in the cytoplasm (see the white line and the intensity profile analysis in Fig. 2B), thus suggesting an abnormal protein localization (Fig. 3B), according to WB results (Fig. 2B).

Data from immunostaining analysis showed no cytoplasmic mislocalization and/or aggregation of FUS protein (Fig. 3C) in all analyzed patients.

Additional immunofluorescence images are reported in Figs S2-S4.

### Mitochondrial morphological changes in LCLs of *SOD1*-, *TARDBP*- and *FUS*-mutated patients

Mitochondria are highly dynamic organelles that constantly fuse and divide in response to diverse stimuli; moreover, their morphology is strictly related to changes in mitochondrial and cellular functions, and an imbalance in mitochondrial dynamics plays a critical role in various neurodegenerative diseases (Itoh et al., 2013; da Silva et al., 2014).

**Fig. 4.**
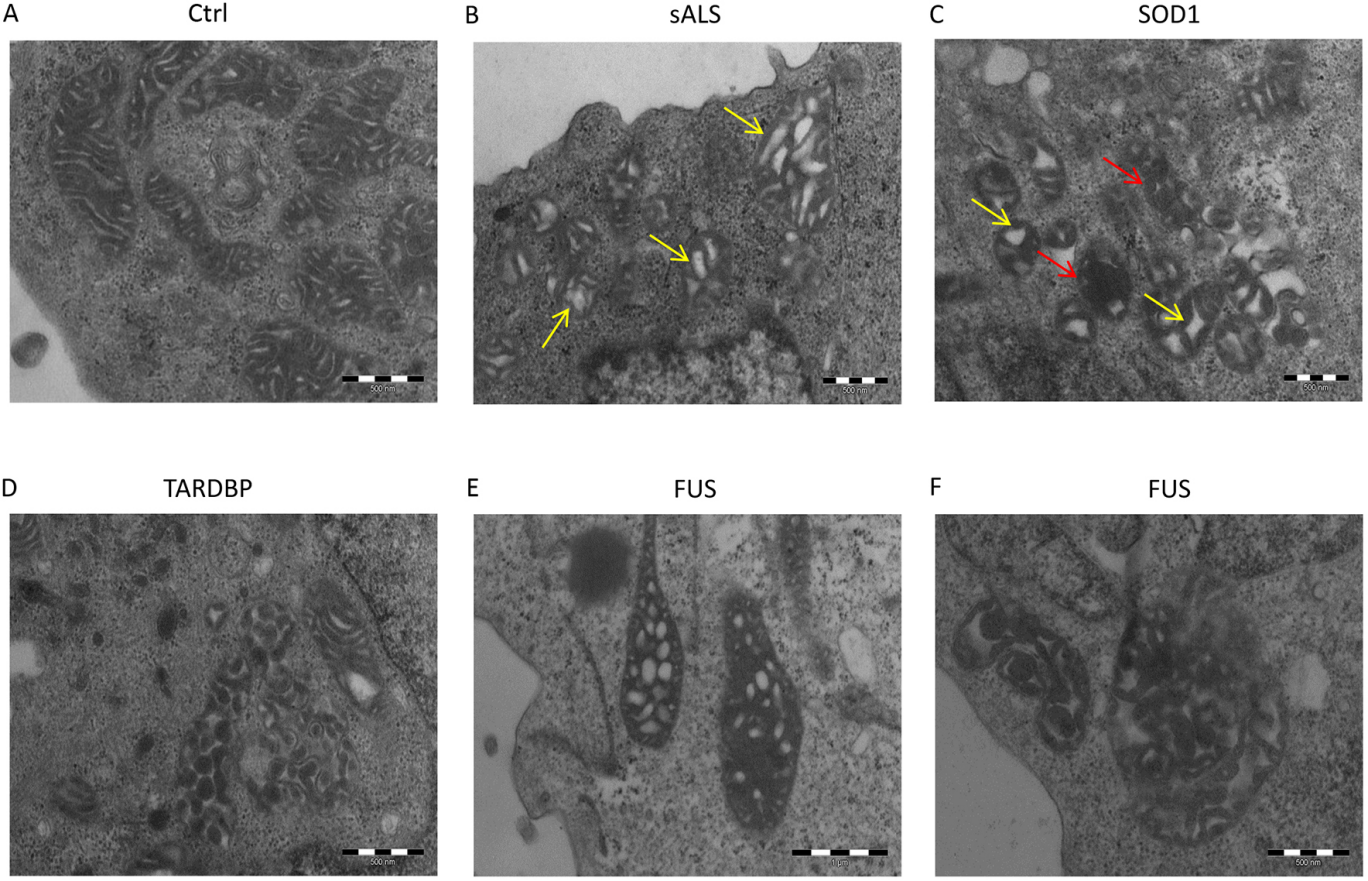
**Mitochondria morphology of Ctrl, sALS, *SOD1*-, *TARDBP*- and *FUS*-mutated LCLs.** Representative images of TEM analysis from Ctrl (A), sALS (B), *SOD1* (C), *TARDBP* (D) and *FUS* (E,F) mutated patients. Ctrl mitochondria were normal, with a longitudinal shape and densely packed cristae (A). Mitochondria from sALS patients were longitudinal with increased vacuolization (yellow arrows) and reduced dimensions (B). Mitochondria from *SOD1*-mutated patients were smaller and round, with increased vacuolization (yellow arrows) and paucity of cristae (red arrows) (C). *TARDBP*-mutated patients showed bigger and round mitochondria with disarranged cristae (D). Mitochondria from *FUS*-mutated patients are completely degenerated: megamitochondria appear in these patients (E,F).

Here, we investigated mitochondrial morphology by means of transmission electron microscopy (TEM). As expected, mitochondria from Ctrl were normal with a longitudinal shape and densely packed cristae (Fig. 4A). sALS patients showed mainly longitudinal mitochondria, although some mitochondria had signs of degeneration, i.e. increased number of vacuoles (see yellow arrows, Fig. 4B) and reduced dimensions (Fig. 4B). In *SOD1*-mutated patients, mitochondria were smaller and mainly round; they presented an increased vacuolization (yellow arrows, Fig. 4C) and cristae are distorted by holes with empty matrix (red arrows, Fig. 4C). *TARDBP*-mutated patients also showed signs of mitochondrial degeneration. Mitochondria are bigger, probably because of a swelling associated with disarrangement of cristae and partial or total cristolysis. The longitudinal shape is lost or severely compromised (Fig. 4D). Finally, in *FUS*-mutated patients, in addition to the signs of degeneration observed in all patients, mitochondria appear giant (megamitochondria) and the regular spacing of cristae is lost (Fig. 4E,F).

Additional TEM images are reported in Fig. S5.

### *SOD1*, *TARDBP* and FUS mutations showed differences in mitochondrial dynamics in LCLs

It is widely accepted that fusion, fission and trafficking may contribute to mitochondrial dysfunction in neurodegenerative diseases (Burté et al., 2015; Itoh et al., 2013). Here, we characterized LCLs according to changes in mitochondrial dynamism, to pinpoint possible mutation-related differences. We analyzed OPA1 and MFN1 as proteins involved in the fusion process, and Drp1 as a protein controlling the fission process.

**Fig. 5.**
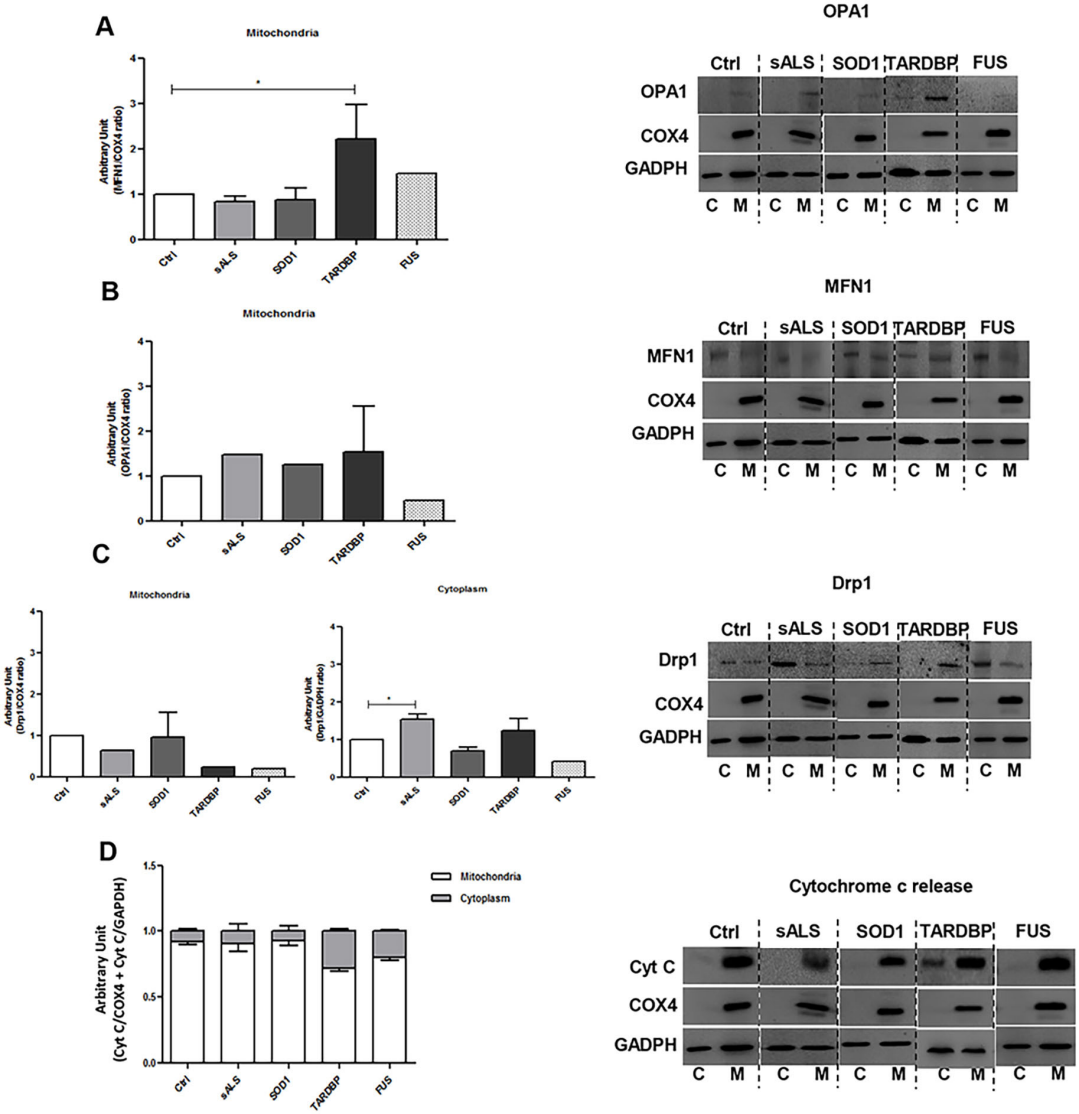
***SOD1*, *TARDBP* and *FUS* mutations showed differences in mitochondrial dynamics in LCLs.** Representative immunoblots for mitochondrial fusion protein (OPA1 and MFN1), and for mitochondrial and cytoplasm fission protein (Drp1), expression levels in Ctrl, sALS, *SOD1*, *TARDBP* and *FUS* LCLs (A-C). COX4 was used for mitochondrial sample normalization; GADPH was used for cytoplasm sample normalization. (D) Representative immunoblot for cytochrome *c* release. The *y*-axis represents the relative amount of cytochrome *c* to either Cox4 (white bars) or GAPDH (grey bars). Densitometric analysis of WB data are performed by ImageJ software. Statistical analysis was carried out on *n*=4 Ctrl, *n*=4 sALS, *n*=3 *SOD1*, *n*=2 *TARDBP* and *n*=2 and *FUS* LCLs. Data are means±s.e.m. and one-way ANOVA followed by Dunnett's multiple comparison test as a *post hoc* test. *P<0.05 in A and C (cytoplasm), all relative to Ctrl. C, cytoplasm; M, mitochondria.

We did not observe differences in OPA1 protein expression levels in the mitochondrial fraction of ALS patients compared to Ctrl (Fig. 5A); of note, *FUS*-mutated patients showed a slight but not significant decrease in OPA1 protein expression levels when compared to Ctrl. MFN1, which, together with OPA1, acts in the fusion process, showed a significant increase in protein expression levels in *TARDBP*-mutated patients (*P*<0.05, Fig. 5B).

Finally, a decrease in Drp1 protein expression levels was reported in the mitochondrial fractions of *TARDBP*- and *FUS*-mutated patients (Fig. 5C). These data agree with TEM results, which showed an altered mitochondrial morphology, i.e. giant or megamitochondria, in *TARDBP*- and *FUS*-mutated patients, which suggests an imbalance of the dynamics versus fusion and an increased connectivity among mitochondria (TEM images: Fig. 4D-F). Moreover, we observed a significant increase in Drp1 protein expression levels in cytoplasm of sALS patients compared to Ctrl (*P*<0.05, Fig. 5C), suggesting an increased mitochondrial fragmentation as evidenced by the presence of small mitochondria (TEM image: Fig. 4C). Moreover, the increase in Drp1 in sALS patients and the reduced dimensions of mitochondria could be associated with an alteration of the mitochondria-associated membranes (MAMs), which regulate mitochondrial dynamism through Drp1 (Polo et al., 2017; Watanabe et al., 2016).

Analysis of the mitochondrial/cytoplasmic partition of cytochrome *c* shows increased mean cytoplasmic level, expressed as a percent of the total cytoplasmic and mitochondrial level of the protein, in *FUS*- and *TARDBP*-mutated samples, whereas no changes were reported in Ctrl, sALS or *SOD1*-mutated samples (Fig. 5D).

### *SOD1*, *TARDBP* and FUS mutations showed differences in mitochondrial respiration and glycolytic flux in LCLs

To assess mitochondrial respiration, the Seahorse XF-24 Bioanalyzer was used to simultaneously measure the oxygen consumption rate (OCR) and the glycolytic flux by measure of the extracellular acidification rate (ECAR) in LCLs of sALS and mutated patients.

#### Oxygen consumption rate (OCR)

**Fig. 6.**
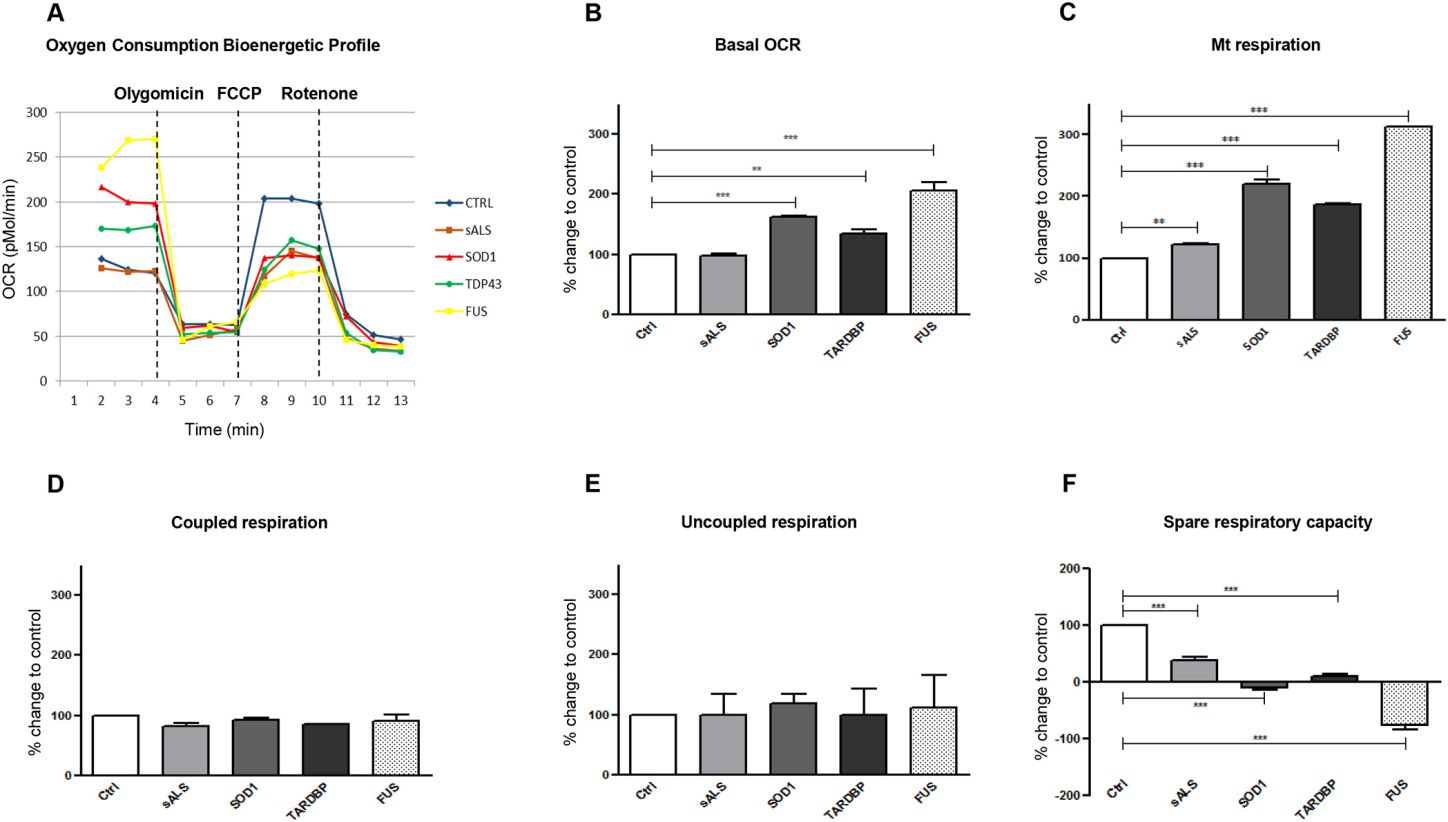
***SOD1*, *TARDBP* and *FUS* mutations showed differences in mitochondrial respiration in LCLs.** Normalized OCR measured by Seahorse XF-24 Bioanalyzer in LCLs of healthy controls, sALS and mutated patients (A). Basal cellular oxygen consumption rate (bOCR) increased significantly (**P<0.01; ***P<0.001) in all patients carrying mutations in *SOD1*, *TARDBP* and *FUS*. sALS patients showed a bOCR like that of Ctrl (B). Mitochondrial oxygen consumption (mOCR) increased significantly in all mutated patients (***P<0.001) and in sALS (**P<0.01) compared to Ctrl (C). No significant differences were reported in ATP-synthase-coupled mOCR and ATP-synthase-uncoupled mOCR (D,E). The spare respiratory capacity (SRC) was diminished in all patients (mutated and sporadic) compared to healthy controls (***P<0.001) (F). Data are means±s.e.m. and one-way ANOVA followed by Dunnett's multiple comparison test as a *post hoc* test.

In Fig. 6A, we reported normalized OCR measurements using the Seahorse XF-24 Bioanalyzer in LCLs of healthy controls, sALS patients and mutated patients. The basal measurements of OCR were recorded before drug injection, then oligomycin, FCCP and rotenone were injected sequentially to measure the key forms of mitochondrial function. Basal cellular oxygen consumption rate (bOCR) increased significantly in all patients carrying mutations in *SOD1*, *TARDBP* or *FUS*, whereas, in sALS patients, bOCR is like that measured in Ctrl subjects (Fig. 6B). To determine whether the increase in OCR was mitochondrial-dependent, we added rotenone, a complex I inhibitor, to LCLs. As reported in Fig. 6C, mitochondrial oxygen consumption rate (mOCR) increased significantly in all mutated patients and in sALS compared to Ctrl. By contrast, no significant differences were reported in both the ATP-synthase-coupled mOCR (i.e. measured after the addiction of oligomycin) and ATP-synthase-uncoupled mOCR (calculated by subtracting mOCR plus oligomycin from mOCR plus rotenone) (Fig. 6D,E). The spare respiratory capacity (SRC), which refers to the mitochondrial ability of cells to generate energy under conditions of great demand, was diminished in all patients (mutated and sporadic) compared to healthy controls (Fig. 6F); of note, SRC reached negative values in *SOD1*- and *FUS*-mutated patients.

#### Extracellular acidification rate (ECAR)

**Fig. 7.**
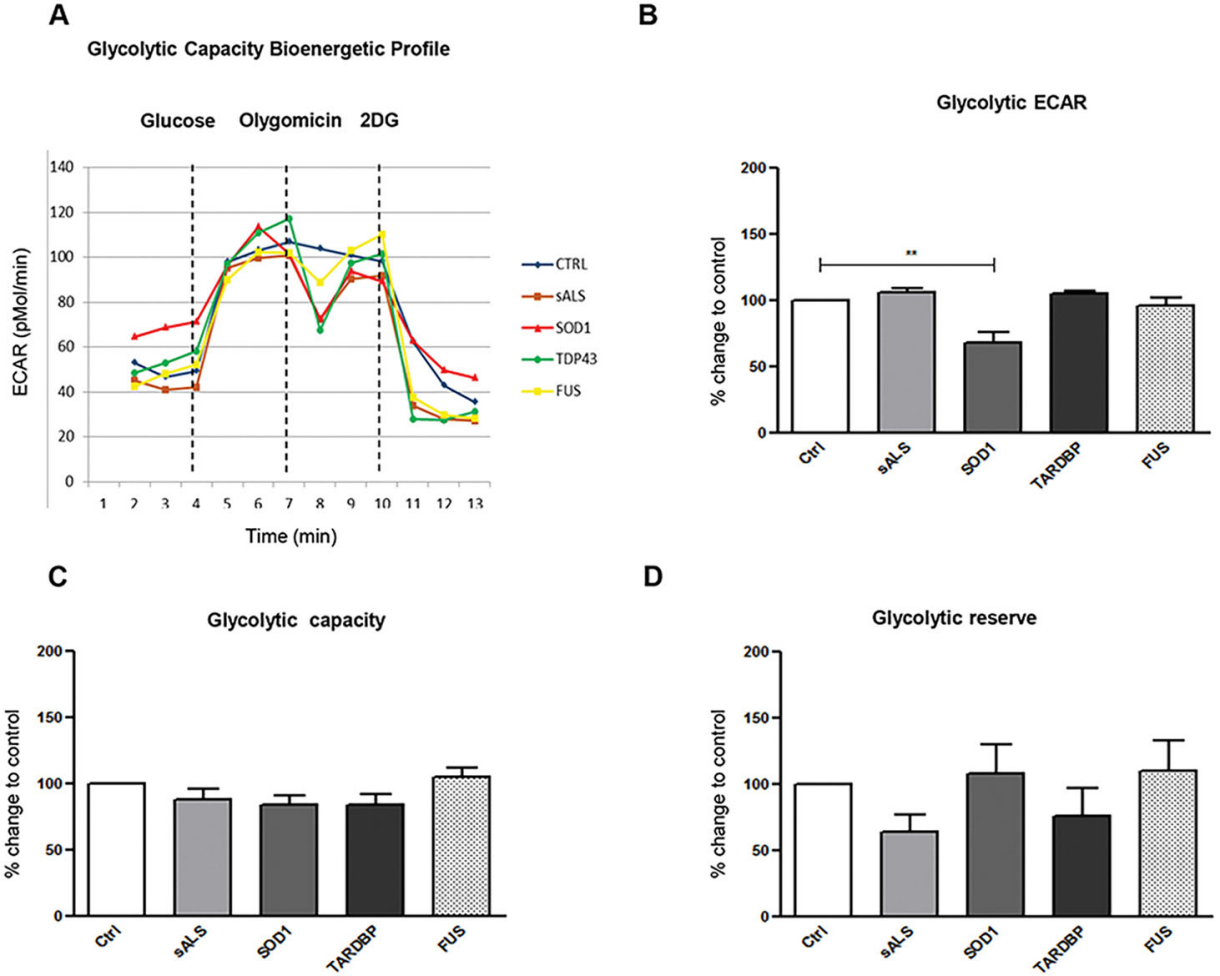
***SOD1*, *TARDBP* and *FUS* mutations showed** **differences in glycolytic flux in LCLs.** Injections of glucose, oligomycin and 2-deoxy-glucose (2-DG) were carried out to determine the glycolytic flux ECAR specific, the glycolytic capacity and the glycolytic reserve between ALS patients and control LCLs, respectively (A). The glycolytic flux ECAR specific was downregulated by about 32% in *SOD1* mutated patients compared with Ctrl (**P<0.01) (B); no significant differences were reported in the glycolytic capacity and glycolytic reserve (C,D). Data are means±s.e.m. and one-way ANOVA followed by Dunnett's multiple comparison test as a *post hoc* test.

Simultaneously to the measurement of OCR, the Seahorse XF-24 Bioanalyzer also analyzes ECAR, an index of cellular glycolytic flux. Subsequent injections of glucose, oligomycin and 2-deoxy-glucose (2-DG) were carried out to determine the glycolytic flux ECAR specific, the glycolytic capacity and the glycolytic reserve in ALS-patient and control LCLs (Fig. 7A). We reported a downregulation of the glycolytic flux ECAR specific (of about 32%) in *SOD1*-mutated patients (Fig. 7B), whereas no significant differences were reported in the glycolytic capacity and glycolytic reserve (Fig. 7C,D).

## DISCUSSION

Peripheral tissues have been progressively gaining the scene in research on neurodegenerative disorders, and peripheral blood has been indicated as a potential source of biomarkers in ALS (Cereda et al., 2013; Gagliardi et al., 2010; Cereda et al., 2006; Nardo et al., 2011). Even though PBMCs may represent a useful tool in the research on ALS pathogenesis to study genetic mechanisms or to unravel whether experimental stress may alter the transcriptional state of the cells, they also display limits. First, PBMCs cannot be grown and stored as a cell line and therefore do not show the right handling flexibility needed for adequate modeling. For this reason, we focused on Epstein-Barr virus (EBV)-immortalized lymphoblastoid cell lines that, while sharing many of the PBMCs' features, can also be grown in culture. Here, we use for the first time LCLs from sALS patients to categorize potential biological molecular signatures associated with the pathology, i.e. protein aggregation and mitochondrial dysfunction. We also take advantage of the availability of LCLs from ALS patients carrying mutations in *SOD1*, *TARDBP* and *FUS* genes to investigate whether and how mutations affect different ALS-linked pathways.

Data from LCLs of sALS patients showed no substantial changes in total protein expression levels of SOD1 and TDP-43, whereas total FUS protein expression levels decreased significantly, thus suggesting the absence of alterations in total protein expression. A different picture appeared when we fractionated nuclear and cytoplasmic compartments. In sALS patients, SOD1, TDP-43 and FUS protein expression levels decreased in both the nucleus and cytoplasm when compared to Ctrl. Nevertheless, no protein relocalization and no signs of protein aggregation were reported. Even if protein mislocalization, particularly TDP-43 and FUS, plays a key role in ALS pathology (Boeynaems et al., 2016; Dormann and Haass, 2011), in LCLs from sALS patients we did not report nucleus-cytoplasmic transport defects and/or protein aggregation. The reason for this could be attributed, at least in LCLs from sALS patients, to the absence and/or low levels of elements, i.e. mutations and nuclear import regulators, which negatively affect the delicate balance of the nuclear import and export pathways. In LCLs from sALS patients, mitochondria maintained a ‘normal’ longitudinal shape but appeared smaller; this could be related to the increased expression of the fission protein Drp1, which helps to create new mitochondria in order to preserve a healthy mitochondrial population (Twig et al., 2008). Finally, OCR measurements did not differ significantly from healthy Ctrl, further indicating that mitochondria from LCLs of sALS patients are only marginally affected by the pathology.

LCLs from *SOD1*-mutated patients showed that, despite an increase in *SOD1* mRNA levels, protein expression levels both in the total soluble fraction and in nuclear/cytoplasm extracts were reduced compared to healthy controls. A similar result was already obtained by our group in PBMCs of sALS patients (Gagliardi et al., 2010), suggesting that either alterations in mRNA stability or the presence of misfolded/aggregated proteins might cause an increase in SOD1 protein expression levels in the insoluble fraction with a concomitant decrease in protein expression levels in the soluble fraction. Mutated SOD1 protein has been reported to form intracellular aggregates that may exert a toxic function (Proctor et al., 2016). In LCLs from *SOD1*-mutated patients, we observed cytoplasm/perinuclear aggregates, suggesting protein recruitment in cytoplasmic aggregates following mutations in the *SOD1* gene. Since aggregates are a hallmark in ALS pathology, the presence of aggregates in *SOD1*-mutated LCLs further corroborates the idea that LCLs could be an interesting device for ALS research. With regards to mitochondrial dysfunction, we did not find significant changes in fusion- and fission-protein expression levels. Nevertheless, TEM analysis revealed alterations in mitochondrial morphology: the presence of smaller and round mitochondria account for favoring the fission over the fusion pathways, as previously observed in cellular and mouse models expressing mutant SOD1 (Magrané et al., 2014). Moreover, the increase in mitochondrial respiration and the decrease in SRC referred to difficulties in the mitochondrial ability of cells to face the increase in energy demand. The altered mitochondrial metabolism was also confirmed by the decrease in the glycolytic ECAR.

In LCLs from patients with mutant *TARDBP*, SOD1, TDP-43 and FUS protein expression levels in RIPA soluble extracts and mRNA expression remained unchanged compared to Ctrl. In fractionated nucleus and cytoplasm, SOD1 maintained its ‘normal’ cytoplasm localization even if its expression was reduced. No changes were also reported in FUS, thus suggesting that the expression levels of these two proteins are not affected by the presence of mutations in the *TARDBP* gene. According to the literature (Oberstadt et al., 2017), in *TARDBP*-mutated LCLs, TDP-43 was depleted from the nucleus and accumulated in the cytoplasm, where the protein formed extranuclear round toxic aggregates (see immunofluorescence images in Fig. 3B). Even if this result needs further and deeper examination, our findings established that LCLs recapitulate ALS pathological features in *TARDBP*-mutated patients, and can be indicated as a compelling model of the disease. Mitochondria from *TARDBP*-mutated patients showed abnormal clustering and elevated levels of MFN1; these findings suggested that mutations in the *TARDBP* gene may alter mitochondrial dynamics; we hypothesize an imbalanced dynamism, with a bias towards the fusion pathway, which is further confirmed by the formation of giant mitochondria containing electrondense globes and vacuoles. However, our results partially disagree with data reported in the literature concerning cultured MNs as well as in the intact sciatic nerve of living mice but also in fibroblasts from patients harboring the A382T mutation in TDP-43, where a fragmented mitochondrial network and impaired mitochondrial transport was reported (Magrané et al., 2014; Onesto et al., 2016; Xu et al., 2010). These discrepancies may be primarily due to the different model adopted: i.e. LCLs from *TARDBP*-mutated patients versus transgenic mouse models or fibroblasts from ALS patients (Magrané et al., 2014; Onesto et al., 2016; Xu et al., 2010). Finally, concerning mitochondrial metabolism, *TARDBP*-mutated LCLs had increased mitochondrial respiration and a reduced SRC, whereas no changes were reported in the glycolytic flux. Our results allow us to speculate that the abnormal mitochondrial morphology could be caused by alterations in components (fusion elements) of the mitochondrial dynamics machinery, whereas intrinsic mitochondrial bioenergetics defects seem to not be involved in this process.

No significant alterations in LCLs of *FUS*-mutated patients, i.e. mRNA up- or downregulation, protein expression levels and protein mislocalization, were reported. We could hypothesize that, in LCLs, FUS mutation is not sufficient to eventually induce a gain and/or a loss of function, which in turn determine protein aberrant localization, as reported by other authors (Ederle and Dormann, 2017; Suzuki and Matsuoka, 2015). What is noteworthy is the presence of megamitochondria, as an index of increase mitochondria damage, thus suggesting a direct effect of FUS mutation on mitochondria.

Finally, augmented levels of cytochrome *c* in the cytoplasm may be suggestive of mitochondrial damage, and the release of cytochrome *c* from the mitochondrion to the cytoplasm is an early step in cellular death via apoptosis (Danial and Korsmeyer, 2004); therefore, apoptotic pathways seem to be recruited both in *FUS*- and *TARDBP*-mutated samples but not in sALS nor in *SOD1* patients.

In conclusion, patient-derived LCLs display features typical of degenerating MNs in ALS, mainly protein aggregation and mitochondrial dysfunction. Consequently, LCLs could be suggested as a valid cellular model of ALS to study specific pathological pathways or possibly to identify new ones. Moreover, LCLs obtained from both sALS and mutated ALS patients have the advantage of expressing physiological levels of mutant genes, thus preventing any trouble due to the over-production of the related protein.

An important aspect arising from this study is how the different gene mutations affect the different analyzed cellular mechanisms. *TARDBP* and *FUS* mutations mainly imbalance mitochondrial dynamism by increasing the fusion process, whereas sALS and *SOD1* mutation mainly affect fission. Thus, at a first glance, we could hypothesize that there are two patient categories: one including patients carrying mutations in RNA-binding proteins, and the other including sALS and SOD1 mutated patients. However, even if *TARDBP* and *FUS* mutations show similarities in terms of mitochondrial dynamism, they are different with regards to protein aggregation and mislocalization. In fact, we showed a similar pathway between *TARDBP* and *SOD1* mutations – the presence of protein aggregation – whereas *FUS* mutation did not induce protein aggregation and/or mislocalization. With regards to mitochondrial metabolism, all LCLs, independently from mutation, are not able to work in a condition of excessive energy request, suggesting that mitochondria from ALS patients are all characterized by a significant metabolic defect. We suggest that morphological differences observed between mutated and sALS patients may account for differences in mitochondrial metabolism. In particular, mitochondrial anomalies reported in sALS patients seem to be limited to mitochondrial morphology, with no evident defect in metabolism according to what is reported in Rodríguez et al. (2012). In mutated patients (*TARDBP* or *FUS*), the increased fusion of mitochondria probably relates mainly to damaged mitochondria. We can suggest that mutations seem to implicate difficulties in handling and/or repair of damaged mitochondria, thus pointing to the observed alteration in mitochondrial functionality.

Finally, it is important to stress that, when analyzing mutated patients with a rare disease, even if the results did not reach statistical significance, we should pay close attention to the obtained data because of the small number of available samples and the importance of each single data point. On the other hand, since patient's blood is relatively easy to obtain and LCLs can easily be settled, it is possible to collect a considerable number of samples specific for each mutation, thus allowing comparison with statistical power between pathological signatures of different mutations, possibly leading to patient stratification on a molecular basis.

## MATERIALS AND METHODS

### Patients’ enrolment

Patients affected by ALS were enrolled at the IRCCS Mondino Foundation, Pavia, Italy. ALS diagnosis was made according to the revised El Escorial criteria (Brooks et al., 2000). Sex- and age-matched healthy volunteers, free from any pharmacological treatment and pathology, were recruited at the Transfusion Centre of the IRCCS Policlinico S. Matteo Foundation in Pavia, Italy. PBMCs from 11 ALS patients and 4 Ctrl were immortalized; of the 11 patients, 4 were sporadic, three harbor mutations in the *SOD1* gene, two harbor mutations in *TARDBP* and two are mutated in the *FUS* gene, as reported in Tables 1 and 2.

**Table 1.**
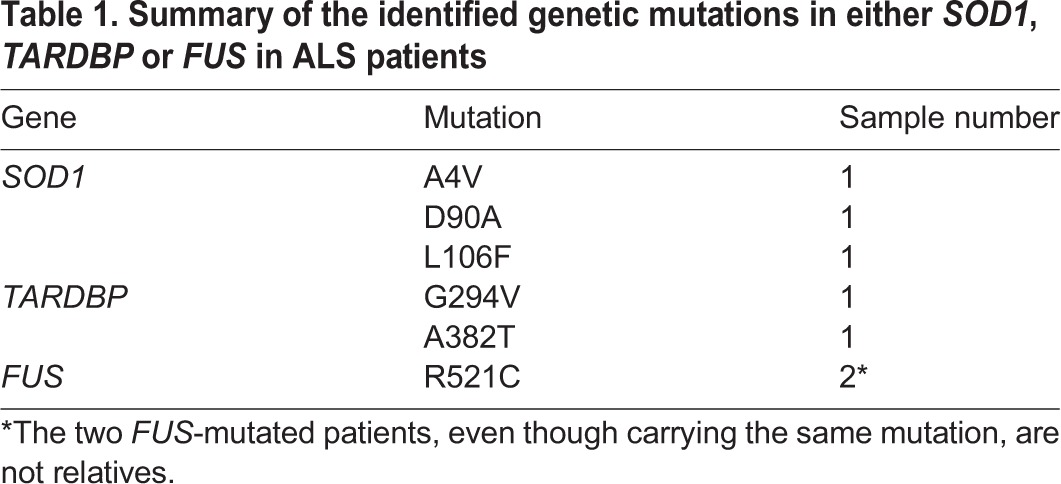
**Summary of the identified genetic mutations in either *SOD1*, *TARDBP* or *FUS* in ALS patients**

**Table 2.**
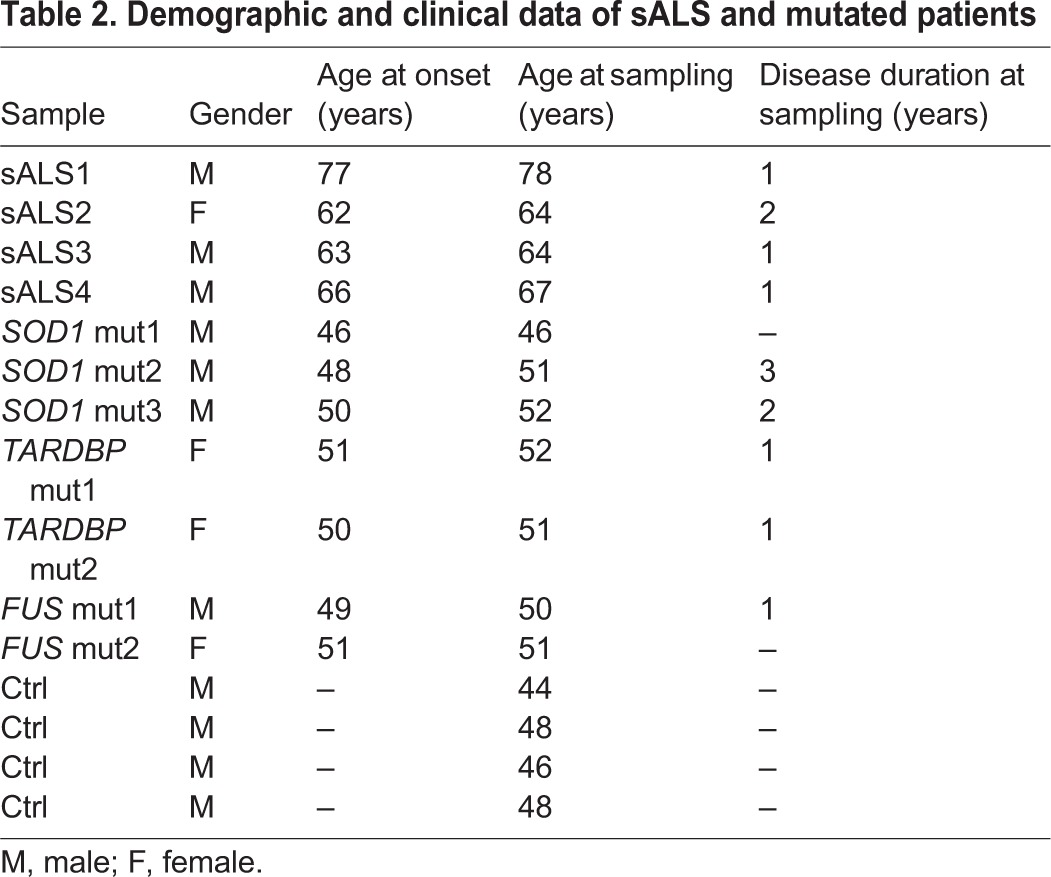
**Demographic and clinical data of sALS and mutated patients**

### EBV immortalization

PBMCs were obtained after informed consent and in accordance with guidelines approved by the Ethical Committee of the ‘C. Mondino’ National Neurological Institute (protocol no. 375/04 – version 07/01/2004). PBMCs were isolated from peripheral venous blood by Histopaque^®^-1077 (Sigma-Aldrich) following the manufacturer's instructions. An aliquot of PBMCs was immortalized into LCLs via EBV infection (Dreser et al., 2017; Scammel et al., 1997, with minor modifications). Briefly, 5×10^6^ cells were re-suspended in RPMI 1640 medium (Sigma-Aldrich), supplemented with 20% fetal bovine serum (FBS; Sigma-Aldrich), 0.3 mg/l L-glutamine, 5% penicillin-streptomycin and cyclosporine A (Sigma-Aldrich). EBV-mix, prepared according to Caputo and collaborators (Caputo et al., 1991), plus RPMI 1640 with cyclosporin A was added to the cells. Cells were incubated at 37°C in a humidified atmosphere with 5% CO_2_ for 1 week. The medium was then changed and cells were left in incubation until clusters of growing cells appeared. Lymphoblastoid cells were maintained and expanded in RPMI 1640 medium, supplemented with 20% FBS, 0.3 mg/l L-glutamine, 5% penicillin-streptomycin, in a humid incubator at 37°C with 5% CO_2_. When required for analysis, cells were pelleted, washed with 1× PBS and further processed as required.

LCL cell viability was assessed by Trypan Blue assay. Briefly, cell suspension was mixed with 0.4% Trypan Blue (Sigma-Aldrich) and counted with the automated cell counter TC20 (Bio-Rad) to evaluate the percentage of live cells, which was about 75-80%.

### Sample preparation

#### RIPA extraction

Total soluble protein samples were extracted in RIPA buffer [50 mM Tris-HCl, pH 8.0, 150 mM NaCl, 1% NP-40, 12 mM deoxycolic acid, supplemented with 1% protease inhibitor cocktail (PIC)]. Protein-containing supernatants were collected and stored at −80°C.

#### Subcellular fractionation

LCL subcellular fractionation was performed according to Schreiber and colleagues (Schreiber et al., 1989), with some modifications. Cells were first re-suspended in an ice-cold hypotonic lysis buffer [10 mM HEPES, pH 7.9, 10 mM KCl, 0.1 mM EDTA, 1 mM dithiothreitol (DTT), 0.5 mM phenylmethylsulfonyl fluoride (PMSF), 1% PIC] and incubated on ice. 10% Nonidet NP-40 (Fluka) was added and samples were centrifuged at the maximum speed (20,000 ***g***). Cytoplasmic proteins were collected and stored at −80°C until use. The pellet was re-suspended in an ice-cold hypertonic nuclear extraction buffer (20 mM HEPES, pH 7.9, 0.4 M NaCl, 1 mM EDTA, 1 mM DTT, 1 mM PMSF fluoride, 1% PIC) and incubated on ice with agitation. The nuclear extracts were centrifuged at the maximum speed and the nuclear proteins were collected and frozen at −80°C until use.

#### Mitochondrial extraction

The mitochondrial fraction was isolated using the Cytochrome C Releasing Apoptosis Assay Kit (Abcam), according to the manufacturer's instructions. Briefly, cells were centrifuged at 600 ***g*** at 4°C, pellets were washed in ice-cold 1× PBS and centrifuged again. Supernatant was discarded and cells re-suspended in 1× Cytosol Extraction Buffer Mix (CEB), plus 1 mM DTT and 1% PIC. Samples were incubated on ice and then centrifuged at 10.000 ***g*** at 4°C. Supernatant was collected as cytosolic fraction. The remaining pellet was re-suspended in Mitochondrial Extraction Buffer Mix (MEB), plus 1 mM DTT and 1% PIC, and stored as mitochondrial fraction.

The protein content of each extracted fraction was then quantified by bicinchoninic acid (BCA) assay (Sigma-Aldrich).

### RNA extraction

Total RNA from sALS and mutated-patient as well as from control-subject cells was extracted with Trizol^®^ reagent (Invitrogen) according to the manufacturer's recommendations. RNA quality and quantity was determined using NanoDrop spectrophotometer (Celbio).

### Reverse transcription

Total RNA was reverse transcribed using the iScript cDNA synthesis kit (Bio-Rad) according to the manufacturer's protocol. The reaction mix was incubated for 5 min at 25°C, for 30 min at 42°C and for 5 min at 85°C. The cDNA samples were stored at −20°C.

### Real-time PCR

SYBR-Green primers were designed with identical annealing and melting temperatures using Primer Express Software (Applera, Italy): SOD1 (fw 5′-GGTCCTCACTTTAATCCTCTATCCAG-3′; rev 5′-CCAACATGCCTCTCTTCATCC-3′); TARDBP (fw 5′-GGTGCAGGTCAAGAAAGAT-3′; rev 5′-GCTCATCTTGGCTTTGCTTAA-3′); FUS (fw 5′-AGCCAGAACACAGGCTATGG-3′; rev 5′-TACCGTAACTTCCCGAGGTG-3′); YWHAZ (fw 5′-CGTTACTTGGCTGAGGTTGC-3′; rev 5′-TGCTTGTTGTGACTGATCGAC-3′), used as housekeeping. The quantitative polymerase chain reaction (qPCR) was run at 95°C for 5 min, 95°C for 15 s and 55°C for 30 s for 40 cycles with a melting curve starting at 55°C and increasing 0.5°C each 30 s. A total of 1 μl of cDNA was used.

### Immunofluorescence

About 1×10^5^ cells were placed on a poly-L-lysine slide (Thermo Fisher Scientific) and incubated at 37°C to allow cell attachment to the slide. Cells were rinsed with 1× PBS and fixed in 4% paraformaldehyde in 1× PBS. Fixed cells were washed with 1× PBS and blocked with 5% normal goat serum in 0.1% Tween-PBS for 1 h. Incubation was performed in blocking solution overnight (ON) at 4°C with the following primary antibodies: rabbit polyclonal anti-SOD1 (sc-11407 Santa Cruz Biotechnologies; dilution 1:100), rabbit polyclonal anti-TARDBP (10782-2-AP Proteintech; dilution 1:50) and rabbit polyclonal anti-FUS (NB100-565 Novus Biological; dilution 1:100). The fluorescent-tagged secondary antibody CFTM 488A goat anti-rabbit (SAB4600036 Sigma-Aldrich; dilution 1:700) was used for detection. Slides were mounted with Prolong^®^ Gold anti-fade reagent with 4′6-diamidino-2-phenylindole (DAPI; Invitrogen) and images were acquired by confocal microscopy (Leica DM IRBE, Leica Microsystems SRL, Italy).

### Western blot analysis

Nuclear, cytoplasmic, mitochondrial and total soluble protein lysates (30 μg) were loaded onto 8-16% SDS-PAGE gel (Bio-Rad). Samples were transferred to nitrocellulose membrane with Trans-Blot^®^ Turbo™ Transfer System (Bio-Rad). After 5% non-fat dry milk blocking, nitrocellulose membranes were incubated ON at 4°C with the following antibodies: rabbit polyclonal anti-SOD1 (sc-11407 Santa Cruz Biotechnology; dilution 1:1000); rabbit polyclonal anti-TARDBP (10782-2-AP Proteintech; dilution 1:1000); rabbit polyclonal anti-FUS (NB100-565 Novus Biological; dilution 1:1000); mouse monoclonal anti-DRP1 (ab56788 Abcam; dilution 1:500); mouse monoclonal anti-Mitofusin1 (ab57602 Abcam; dilution 1:500); rabbit monoclonal anti-OPA1 (ab157457 Abcam; dilution 1:1000); mouse monoclonal anti-Cytochrome c (ab65311 Abcam; dilution 1:200). Rabbit polyclonal anti-H1 (sc-67324 Santa Cruz Biotechnology; dilution 1:250) and mouse monoclonal anti-lactate dehydrogenase (LDH; sc-133123 Santa Cruz Biotechnology; dilution 1:1000) were used as nuclear and cytoplasm loading control, respectively; mouse monoclonal anti-GAPDH (sc-47724 Santa Cruz Biotechnology; dilution 1:1000) was used as total soluble loading control, and mouse monoclonal anti-COX4 (ab110261 Abcam; dilution 1:1000) was used as mitochondrial loading control. Immunoreactivity was detected using donkey anti-rabbit (NA9340) or anti-mouse (NA931) secondary peroxidase-conjugated antibody (GE Healthcare; dilution 1:10,000). ECL Select (GE Healthcare) was used for chemiluminescence detection. Densitometric analysis of the bands was performed using ImageJ software (http://rsb.info.nih.gov/ij/).

### TEM sample preparation

Approximately 3×10^6^ cells were washed in 1× PBS and incubated with the fixing solution (2.5% glutaraldehyde and 2% paraformaldehyde in cacodylate buffer, pH 7.3) for 4 h at 4°C, followed by a post-fixation in 1.5% osmium tetroxide for 1 h at room temperature, and Epon-Araldite embedding (Necchi et al., 2010). Ultrathin sections (∼70 nm thick) were cut from the resin blocks and stained with uranyl acetate/lead citrate, and observations were performed using conventional electron microscopy at Centro Grandi Strumenti, Università degli Studi di Pavia, Italy.

### ECAR and OCR

The detection of the ECAR (indicator of glycolysis) and of the OCR (indicator of cellular respiration) was performed using the Seahorse XF24 Bioanalyzer (Seahorse Bioscience-M&M Services SRL). The measurement of ECAR allows the evaluation of the glycolytic flux via lactate production. Initially, ECAR readings were obtained in the absence of glucose to determine the non-glycolytic ECAR contribution to total basal ECAR. Subsequent injections of glucose, oligomycin and 2-deoxyglucose (2-DG) were performed to determine the glycolytic flux, the cellular maximum glycolytic capacity and the glycolytic reserve, respectively. As well as measuring ECAR, the Seahorse XF24 Bioanalyzer also measures OCR by the addition of the complex I inhibitor rotenone, the coupling efficiency by the addition of oligomycin, an ATP synthase inhibitor, and the spare respiratory capacity (i.e. the ability of the energy substrate supply and oxidative phosphorylation to face increased energy demand) by the addition of FCCP, which uncouples oxidative phosphorylation from ATP synthesis.

### Statistical analysis

All the experiments were performed at least three times. Values were expressed as means±s.d. Statistical analysis was performed by one-way analysis of variance (ANOVA) followed by Dunnett's multiple comparison test as a *post hoc* test (GraphPad Prism version 5, San Diego, CA, USA). Values were considered statistically significant when *P*-values were <0.05.

## Supplementary Material

Supplementary information
