# Supplementary material for: Lymphoblastoid cell lines as a model to understand amyotrophic lateral sclerosis disease mechanisms
Source: Dis Model Mech. 2018 Mar 1;11(3):dmm031625. doi: 10.1242/dmm.031625 (PMC5897724; doi:10.1242/dmm.031625)
Supplement: Supplementary information [file dmm-11-031625-s1.pdf]

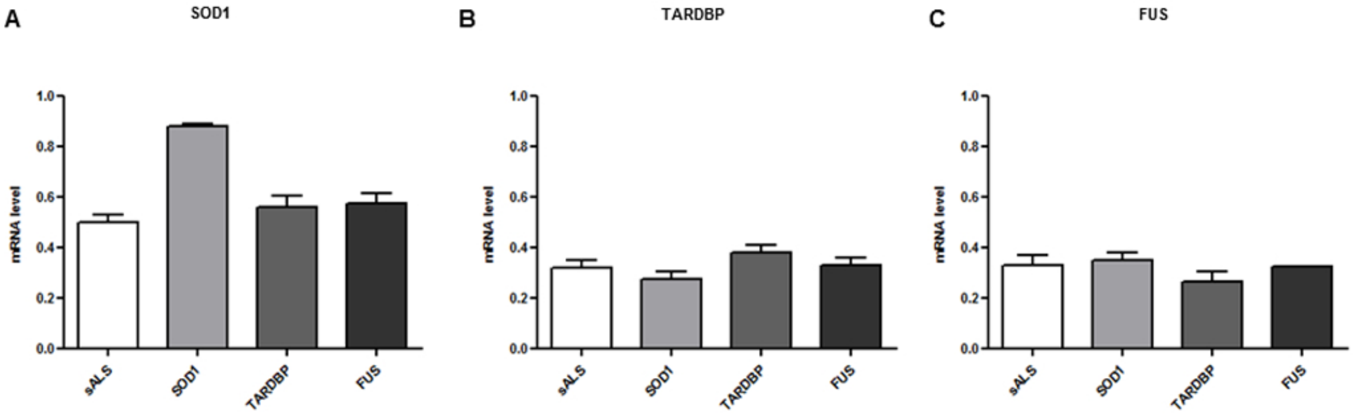

Fig. S1

Fig. S1. mRNA levels in LCLs of sALS and mutated patients.

SOD1 mRNA increased significantly in SOD1 mutated patients (A), while no differences were reported for TARDBP and FUS mutations (B, C).

Disease Models & Mechanisms • Supplementary information

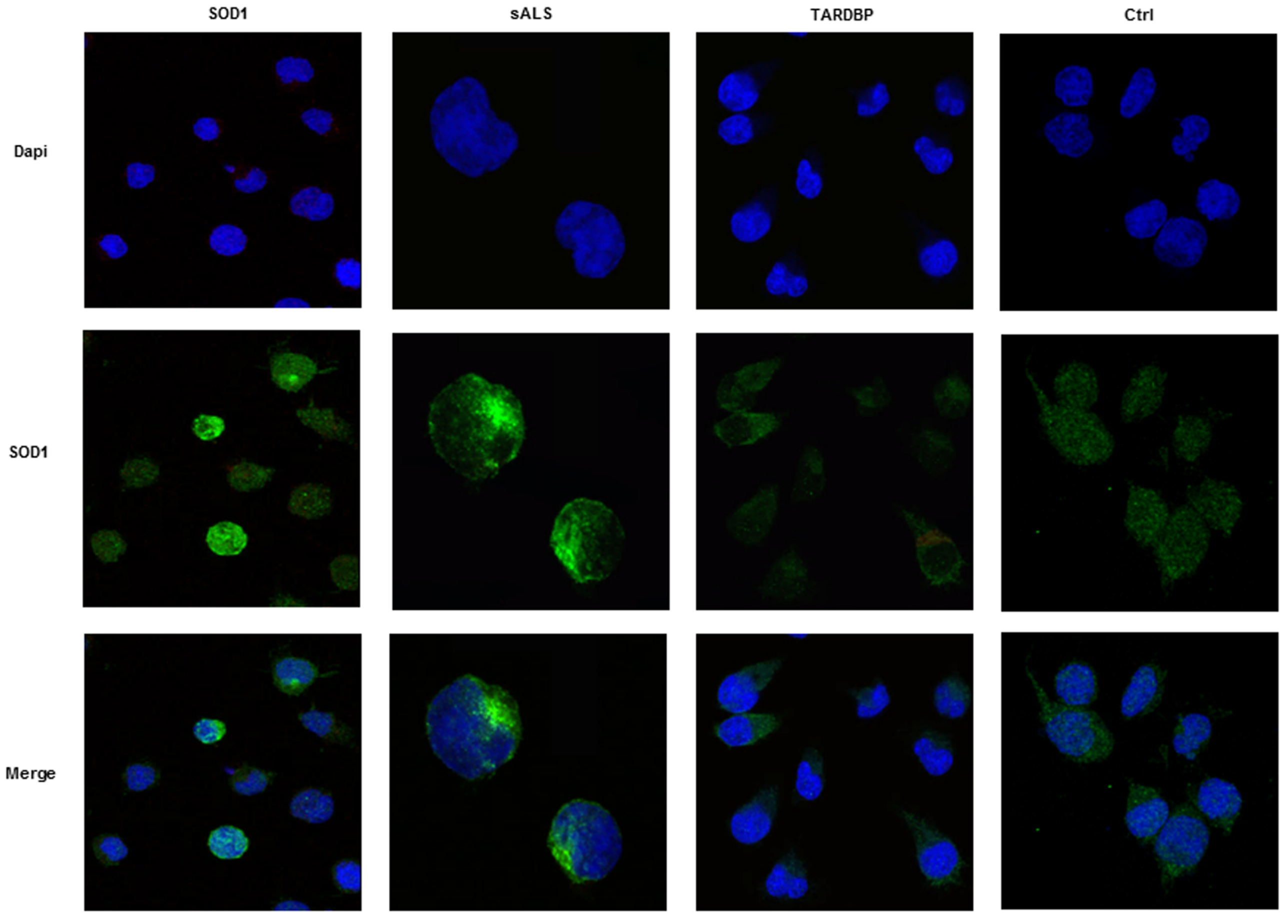

Fig. S2

Fig. S2. SOD1, TARDBP and FUS mutations showed protein re-localization and aggregation in LCLs. Additional representative immunostaining of SOD1 in LCLs of sALS, SOD1, TARDBP and FUS mutated patients.

Disease Models & Mechanisms • Supplementary information

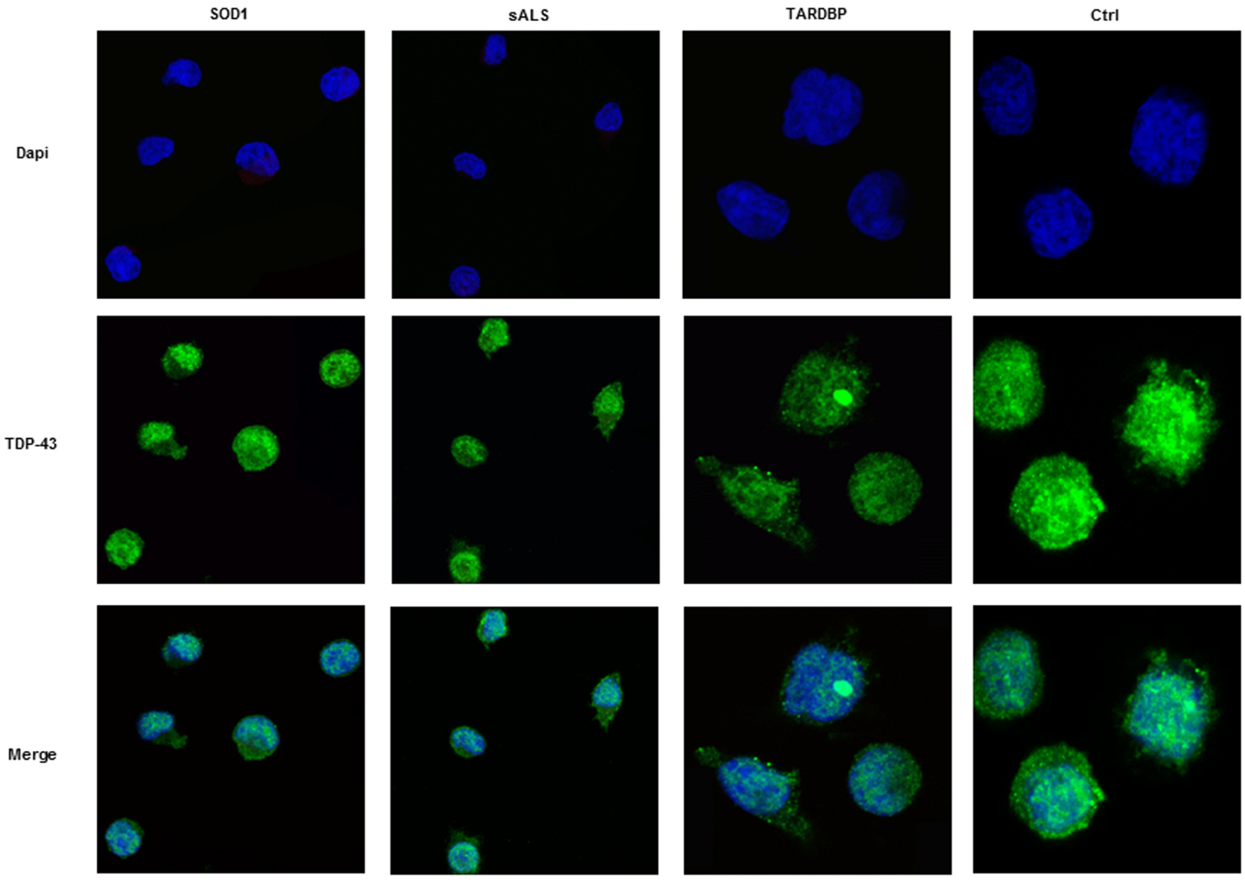

Fig. S3

Fig. S3. SOD1, TARDBP and FUS mutations showed protein re-localization and aggregation in LCLs. Additional representative immunostaining of TDP-43 in LCLs of sALS, SOD1, TARDBP and FUS mutated patients.

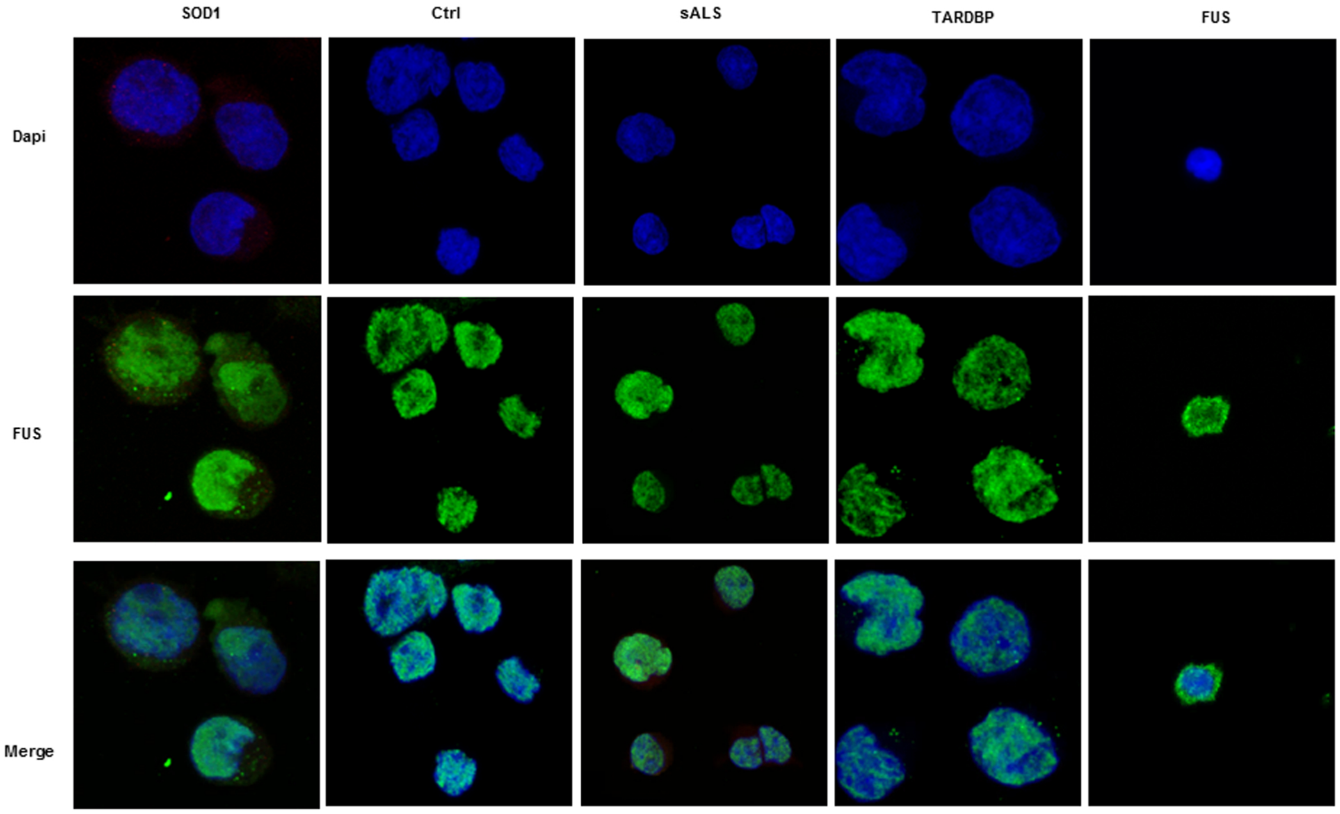

Fig. S4

Fig. S4. SOD1, TARDBP and FUS mutations showed protein re-localization and aggregation in LCLs.

Additional representative immunostaining of FUS in LCLs of sALS, SOD1, TARDBP and FUS mutated patients.

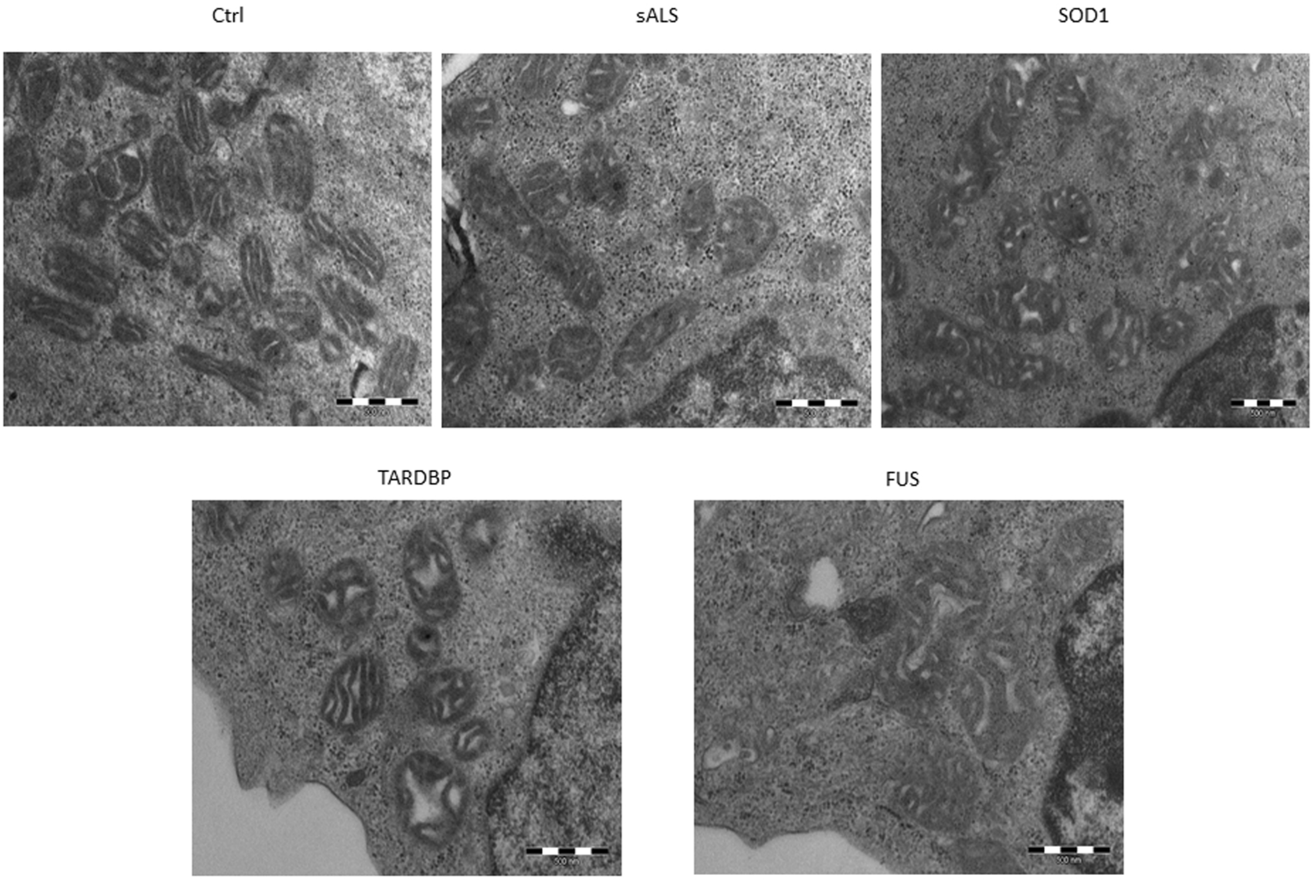

Fig. S5

Fig. S5. Mitochondria morphology of Ctrl, sALS, SOD1, TARDBP and FUS mutated LCLs.

Additional representative images of transmission electron microscopy analysis from Ctrl, sALS, SOD1, TARDBP and FUS mutated patients.
